# Agricultural matrices affect ground ant assemblage composition inside forest fragments

**DOI:** 10.1371/journal.pone.0197697

**Published:** 2018-05-23

**Authors:** Diego Santana Assis, Iracenir Andrade Dos Santos, Flavio Nunes Ramos, Katty Elena Barrios-Rojas, Jonathan David Majer, Evaldo Ferreira Vilela

**Affiliations:** 1 Departamento de Entomologia, Universidade Federal de Viçosa, Viçosa, Minas Gerais, Brazil; 2 Centro de Formação Interdisciplinar Universidade Federal do Oeste do Pará, Santarém, Pará, Brazil; 3 Instituto de Ciências da Natureza, Universidade Federal de Alfenas, Alfenas, Minas Gerais, Brazil; 4 School of Biological Sciences, University of Western Australia, Perth, Australia; 5 School of Molecular and Life Sciences, Curtin University, Perth, Australia; University of Sydney, AUSTRALIA

## Abstract

The establishment of agricultural matrices generally involves deforestation, which leads to fragmentation of the remaining forest. This fragmentation can affect forest dynamics both positively and negatively. Since most animal species are affected, certain groups can be used to measure the impact of such fragmentation. This study aimed to measure the impacts of agricultural crops (matrices) on ant communities of adjacent lower montane Atlantic rainforest fragments. We sampled nine forest fragments at locations surrounded by different agricultural matrices, namely: coffee (3 replicates); sugarcane (3); and pasture (3). At each site we installed pitfall traps along a 500 m transect from the interior of the matrix to the interior of the fragment (20 pitfall traps ~25 m apart). Each transect was partitioned into four categories: interior of the matrix; edge of the matrix; edge of the fragment; and interior of the fragment. For each sample site, we measured ant species richness and ant community composition within each transect category. Ant richness and composition differed between fragments and matrices. Each sample location had a specific composition of ants, probably because of the influence of the nature and management of the agricultural matrices. Species composition in the coffee matrix had the highest similarity to its corresponding fragment. The variability in species composition within forest fragments surrounded by pasture was greatest when compared with forest fragments surrounded by sugarcane or, to a lesser extent, coffee. Functional guild composition differed between locations, but the most representative guild was ‘generalist’ both in the agricultural matrices and forest fragments. Our results are important for understanding how agricultural matrices act on ant communities, and also, how these isolated forest fragments could act as an island of biodiversity in an ‘ocean of crops’.

## Introduction

In many parts of the world, agricultural practices are the main causes of deforestation and forest fragmentation [1]. Fragmentation occurs when a forest area is cleared with the objective of using it for the establishment of crops, settlements or highways [2]. The result of this action is the division of the forest into discontinuous patches or fragments of native vegetation, enclosed by agricultural crops (matrices) [2,3], a situation that can affect forest dynamics in various ways (reviewed by Fahrig [4]).

The impact of fragmentation on forest species emerges from two components: the reduction of area (habitat loss per se) and the isolation between the remaining habitat fragments [3,4]. Both of these can cause positive and negative consequences to forest dynamics [4]. Generally, when the fragments are closer, the easier movement of species between them could reduce the risk of extinction [2]. On the other hand, when the fragments are distant from each other the lack of connectivity exacerbates the effects of habitat loss [4,5], reductions in environmental services [6–8], reductions of resources, and soil degradation [9]. For animal species specifically, these could result in increases in intra- and interspecific competition [3,10], changes in behavior [10], loss of foraging and nesting sites [11], the extinction of specialized species [2,11] and changes in functional guild composition [12,13]. These negative consequences are some of the largest ecological problems threatening biodiversity today [3,14,15].

The nature of the surrounding agricultural matrices affects forest species dynamics [2] at local, landscape and regional scales [16]. Its degree and type of influence on arthropod assemblages can depend on the percentage of forest remaining in the landscape [17] and the remnant’s area, edge and shape [18]; conversely, assemblages in the matrix can be influenced by the distance of the matrix from the forest [19]. Agricultural land is inhospitable to most forest species [9,11] and such matrices exert pressure on forest fragments [20,21], working as a biodiversity filter, which affects the movement of species between and within fragments [9]. They may also exert negative influences by altering components of the food web [22], homogenizing the community [23], and facilitating the spread of exotic and invasive species [24].

Forest fragmentation also generates a transitional area (edge) between the matrix and the forest areas. The abiotic characteristics of this zone are different and could in turn generate several “edge effects” [2,25]. These edge effects affect the microclimate, structure, biodiversity and ecological function of the forest fragment [26,27]. They could also influence the interactions between species found in the fragments [28]. The incidence of these edge effects depends on the time since fragmentation, the shape and area of the fragment and also of the nature of the surrounding land management [29]. During the first years after fragmentation the edges experience microclimatic changes that may cause changes inside the forest fragment [29]. Later, with the passing of years, the edge could act in two main ways depending on the land management around it [19]. When the nature of the surrounding matrices is more benign, the edge vegetation may regenerate and act as a buffer to abiotic factors present in the matrix, such as wind and solar radiation, both of which could potentially affect the interior of a forest fragment [30]. However, in cases where the matrix around the fragment is heavily exploited, the regeneration of the edge is slower or non-existent and, as a consequence, there is a larger influence of the surroundings on the fragment interior [29].

In general, to measure the effects of forest fragmentation a variety of organisms can be used [31,32] and their response can be numeric or functional [32]. Arthropods are an excellent group to use as bioindicators of forest fragmentation effects [33]. Because of their richness and abundance, there are plenty of taxa to work with and they are distributed in almost every habitat [31,34]. Arthropod communities also play an important role in ecological services, including pollination, seed dispersal, and pest limitation [31,35]. Consequently, changes in arthropod communities often have consequences in these functions [31,33]. An alteration in arthropod communities could therefore adversely modify the environment and its basic functions [8], including important environmental services.

Among the arthropods, ants are a group that are commonly used as bioindicators [36], especially in land management. Their use results from their abundance [37], number of species, their varied ecology [36], their ease of sampling and their important role in the environment [33]. Ant species have been subdivided in several functional groups or “guilds”, based on their general ecology, food preferences, behavioral characteristics and nesting habits [38,39]. Information on ant functional guilds that occur in an area could be used to reinforce other impact-measuring procedures, such as species composition, because it reflects differences in ant assemblages from a functional point of view [39].

The focus of the current study was to elucidate how ant alpha-diversity (i.e. species richness), species composition and functional guild composition of ground ants in fragments of lower montane Atlantic rainforest in southeastern Brazil are influenced by different types of agriculture in order to better understand how the nature of forest fragmentation influences such communities. We also considered whether all matrices of a particular crop influence the ant fauna to the same degree. We expected that agricultural matrices would have fewer species of ants than forest fragments and that the similarities in species composition among the same agricultural matrices would be greater than among the remaining forest fragments. We also expected that the predominant functional guilds in the forest fragments would be predators, while in the agricultural matrices they would be generalists.

## Materials and methods

### Study site

The work was conducted in three regions, Alfenas, Areado and Serrania, which comprise the Alfenas microregion in southern Minas Gerais State, Brazil (21°25’45”S, 45°56’50”W) (Table 1). The Alfenas microregion is a transitional area between semideciduous Atlantic Forest and Cerrado (Brazilian savanna) [40]. This area has an annual average temperature of 23°C, and precipitation of 1600 mm, with a mean relative humidity of 70% and elevation ranging from 720 to 1,350 m [41].

**Table 1:** Type of agricultural matrix surrounding the forest fragment, popular name of the fragment in the region, total size and geographic coordinate of sampled forest fragments.

Fragment surrounded by:	Popular Name	Area (ha)	Latitude	Longitude
Pasture	N	24.8	S 21°28'07"	W 46°09'46"
Pasture	São Tomé	49.0	S 21°28'14"	W 45° 59' 20"
Pasture	Matão	20.9	S 21°30'16"	W 45°52'38"
Sugarcane	M	56.1	S 21°27'24"	W 46°10'07"
Sugarcane	Porto	87.2	S 21°25'16"	W 46°07'22"
Sugarcane	I	37.1	S 21°25'35"	W 46°05'39"
Coffee	Caiana	26.3	S 21°35'59"	W 45°55'10
Coffee	Paraíso	36.9	S 21°21'46"	W 45°50'26"
Coffee	Cemitério	23.0	S 21°33'34"	W 45°56'15"

The Alfenas microregion is occupied by permanent cultures, the most common being: pastures (51%), coffee (17%), and rotating cultures like sugarcane and maize (7%) [42]. The native forest is represented by numerous non-contiguous patches (fragments) that constitute approximately 9% of the total area. We defined a sample area in each region and for the purposes of this paper we refer to these as ‘locations’. The location in strict sense is the forest fragment plus its surrounding agricultural matrix. The types of matrices we investigated were coffee (*Coffea arabica*), pasture (*Brachiaria sp*.) and sugarcane (*Saccharum officinarum*). The fragments studied were all preserved remnants of lower montane Atlantic rainforest [43]. All agricultural matrices were in direct contact with the forest fragments. In total, there were nine locations, three of each agricultural matrix and their respective adjacent forest fragments.

### Ant sampling

We used pitfall traps of 13.8 cm internal diameter and 9 cm of depth, containing 200 ml preservative solution (water, salt and detergent), to sample the ground ant community on each location. This is a conventional method for sampling ground arthropods that is commonly used worldwide [39]. An investigation that compared the efficiency and cost of sampling methods for arthropods, [44] indicated that pitfall traps are the best in terms of cost-benefit.

**Fig 1:**
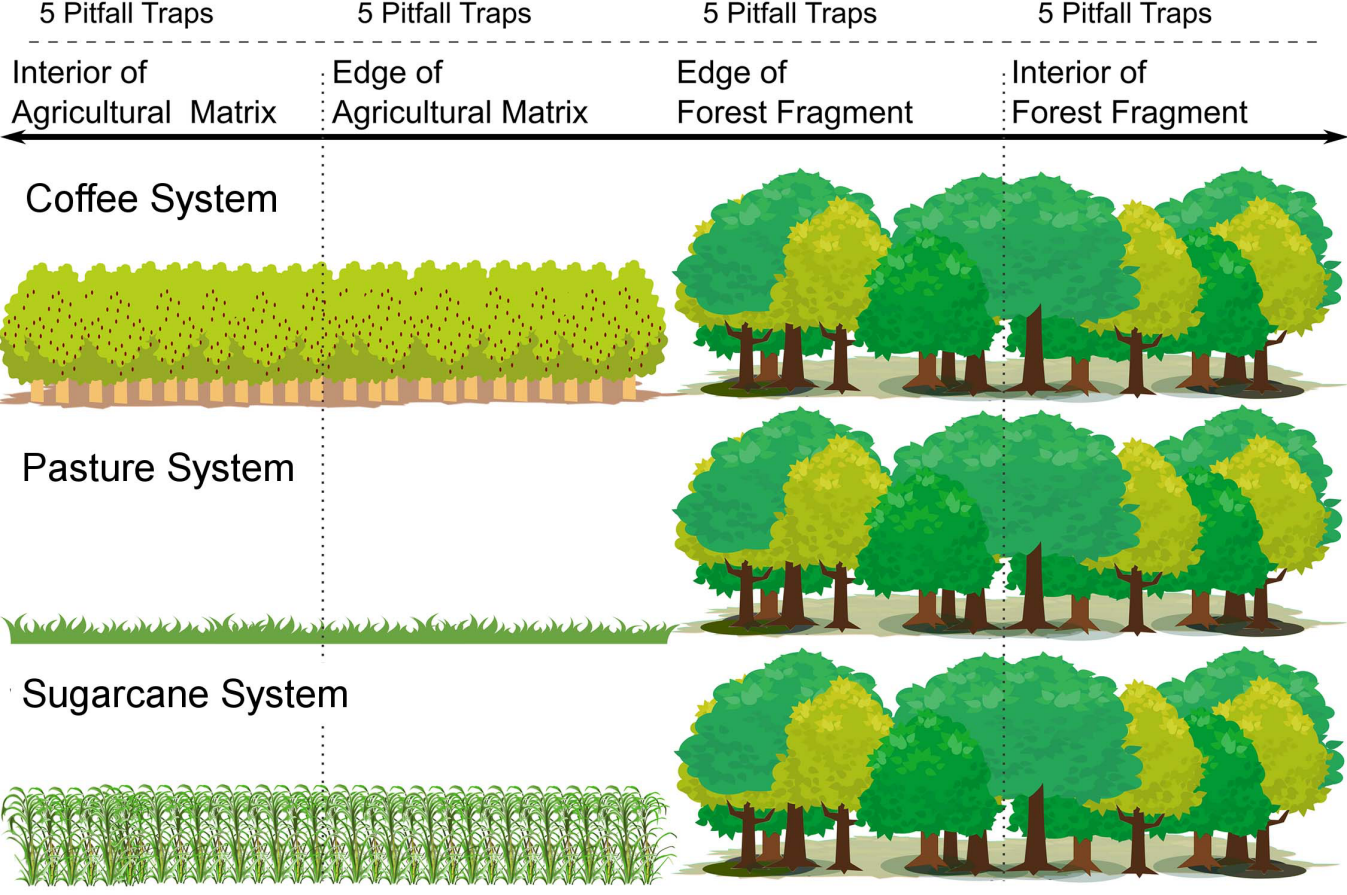
Distance gradient in each system. Each system has three replicates, with five pitfall traps for each place in the system (interior of agricultural matrix, edge of agricultural matrix, edge of forest fragment and interior of forest fragment).

The sampling was performed during the period March—May, 2011 (end of rainy season through to beginning of dry season). First, we set up a 500 m transect in each location. The transect ran from the interior of the matrix to the interior of the forest fragment (Fig 1). We installed a total of 180 traps, i.e. 20 traps in each transect, with a 25 m separation between each trap. The traps were subdivided into four groups or ‘categories’ according to distance as follows: (1) interior of agricultural matrix (0–100 m); (2) edge of agricultural matrix (125–225 m); (3) edge of forest fragment (250–350 m); and (4) interior of forest fragment (375–475 m) (Fig 1). The edges of the agricultural matrices were in direct contact with the edges of forest fragments. We consider only the first 150 m of the forest fragment as the fragment edge. From 150 m onwards the adjoining agricultural matrix has a lower influence on the forest fragment [45]. The traps were maintained for 48 h in the field. There were instances when traps were broken (nine traps in the total); in these situations we replaced them with new ones in the same point, and maintained them for a further 48 h.

The ants collected were identified using Bolton’s identification keys [46,47], AntWeb [48] and personal collection of Iracenir Andrade Dos Santos. The voucher material is archived at the Universidade Federal de Alfenas, Universidade Federal do Oeste do Pará and in the Museum Paraense Emílio Goeldi. All trapping was performed with the permission of land owners.

### Statistical analysis

The total number of species per location in fragments and matrices were first compiled and totals for each matrix type were also summed. Rarefaction curves were then performed in order to analyze if our sample effort was efficient at assessing the richness in the locations. We used the data of incidence of species data in the locations to build the curves, and used the function ‘rarecurve’ from the vegan package v2.4.4 [49] to calculate the rarefaction curves.

We created one generalized linear mixed models (GLMM) using the software R [50] and the package ‘*lme4’* [51], and one generalized linear model (GLM), then we tested the residual errors by the weight of AIC using the package ‘bbmle’ [52]. The best-fit model was a negative-binomial error family (our data were overdispersed). In the first model, GLMM, ant species richness, i.e. the number of collected species, was the response variable and the agricultural matrix and forest fragment type were the explanatory variables; as the random variable we used the sample location. We also performed the Tukey post-hoc test, and the results were separated into three groups of comparisons: 1) differences among forest fragments; 2) differences among agricultural matrices, and; 3) differences among forest fragments and the agricultural matrix in direct contact with it. In the second model, the response variable was also ant species richness and the explanatory variables were category (interior of agricultural matrix, edge of agricultural matrix, edge of forest fragment and interior of forest fragment) in the each agricultural matrix and forest fragment type. In addition, we tested the pairwise difference among species richness using the function ‘glht’ from the package multcomp v.1.4.6 [53], using Tukey contrasts, using the p value correction for false discovery rates, ‘fdr’ [53].

To visualize the arrangement of the ant communities, we used non-metric multidimensional scaling (nMDS) of pitfall trap species counts. To do this, the species matrix was changed to presence/absence and relationships between sites calculated using Jaccard’s similarity index [54]: C_j_ = a/(a + b + c), where a is the number of species held in common and b and c are the number of species at the two sites under comparison. The nMDS ordination analyses then places the more similar communities together in space, and the less similar ones further apart [55]. We chose this analysis because it preserves the similarity relationship among the communities [55].

We then performed a PERMANOVA [56] and a PERMDISP [57]. The PERMANOVA analysis is a non-parametric test for a multivariate hypothesis of difference of compositional species among groups [56]. This test is based on a similarity matrix and can partition the variation among the individuals according an ANOVA model [56,58]. To ecological data is generally highly skewed so other tests such as MANOVA cannot be used, because the assumption of those tests is not true for this ecological dataset [56]. We used the function ‘adonis2’ from the vegan package v2.4–4 [49] and we based the analysis on 9999 permutations. The PERMDISP analysis was applied to test the homogeneity of dispersion in our dataset; for this we used the function ‘betadisper’, also from vegan package v2.4–4 [49]. The ‘betadisper’ function performs a test for homogeneity of dispersion analogous to Lavene’s test. The test also computes a *pseudo*-*F*-statistic to compare the centroids derived from the similarity measure of biodiversity [57]. This test was also performed as a post-hoc test to the PERMANOVA to identify if the dispersion of the multivariate data was interfering with the PERMANOVA results [57]. PERMDISP analysis does not identify the shape of the ‘cloud’ data, rather, this analysis can only detect their relative spread [58]. Both PERMANOVA and PERMDISP computes a *pseudo*-F; *pseudo*-F is different from Fisher’s F ratio. This difference is because we expected a non-normal distributed individual variable [56].

To compare the functional guilds in the localities we used the classification proposed by Delabie et al. [38] with some modifications as follows: Attina (fungus-farming ants such as *Atta*), predators (dominant, generalist and specialist), generalists (dominant, generalist and opportunistic), army ants and arboreal ants. Some ants in this paper belong to more than one group in Delabie's classification. To test the difference in the proportion of functional guilds in the sampled sites we used the G Test [59] This compares the values of the categories with the expected values based on probability they be equal, i.e., all locations have the same number of species in the functional guilds. To undertake this we used the function ‘G.test’ from the package ‘RVAideMemoire’ [60].

## Results

We collected 21,136 ants, belonging to 181 morphospecies and 40 genera (S1 Table). The forest fragments had 14,492 individuals and 133 species, and the matrices had 6,644 individuals and 121 species. There were examples from eight subfamilies: Myrmicinae, Formicinae, Dorylinae, Dolichoderinae, Ponerinae, Proceratinae, Ectatomminae and Pseudomyrmicinae. The richest genus in the samples was *Pheidole* (Myrmicinae), with 38 morphospecies, and the most abundant genus was *Atta* (Myrmicinae), with 5,630 individuals.

The number of species sampled in each location is shown in Table 2, along with the total species sampled when locations of each crop type are combined. There was a large variation in number of species sampled, with totals ranging from 10–57 in the fragments and 21–35 in the matrices. The rarefaction curves for each type of fragment and matrix indicated that our sampling provided a good coverage of the species present in these areas; the apparently low coverage of species in the pasture matrix was probably an artifact of the low number of individuals caught (S1 Fig).

**Table 2:** Numbers of species of ants trapped in fragments and matrices at the various locations, and also the total species trapped when like-locations are combined. (FF–forest fragment, AM–agricultural matrix).

	FF Sugarcane	FF Pasture	FF Coffee	AM Sugarcane	AM Pasture	AM Coffee
Location 1	57	28	28	34	35	34
Location 2	37	10	24	21	24	24
Location 3	21	28	29	25	26	32
All locations	76	52	59	57	52	60

**Fig 2:**
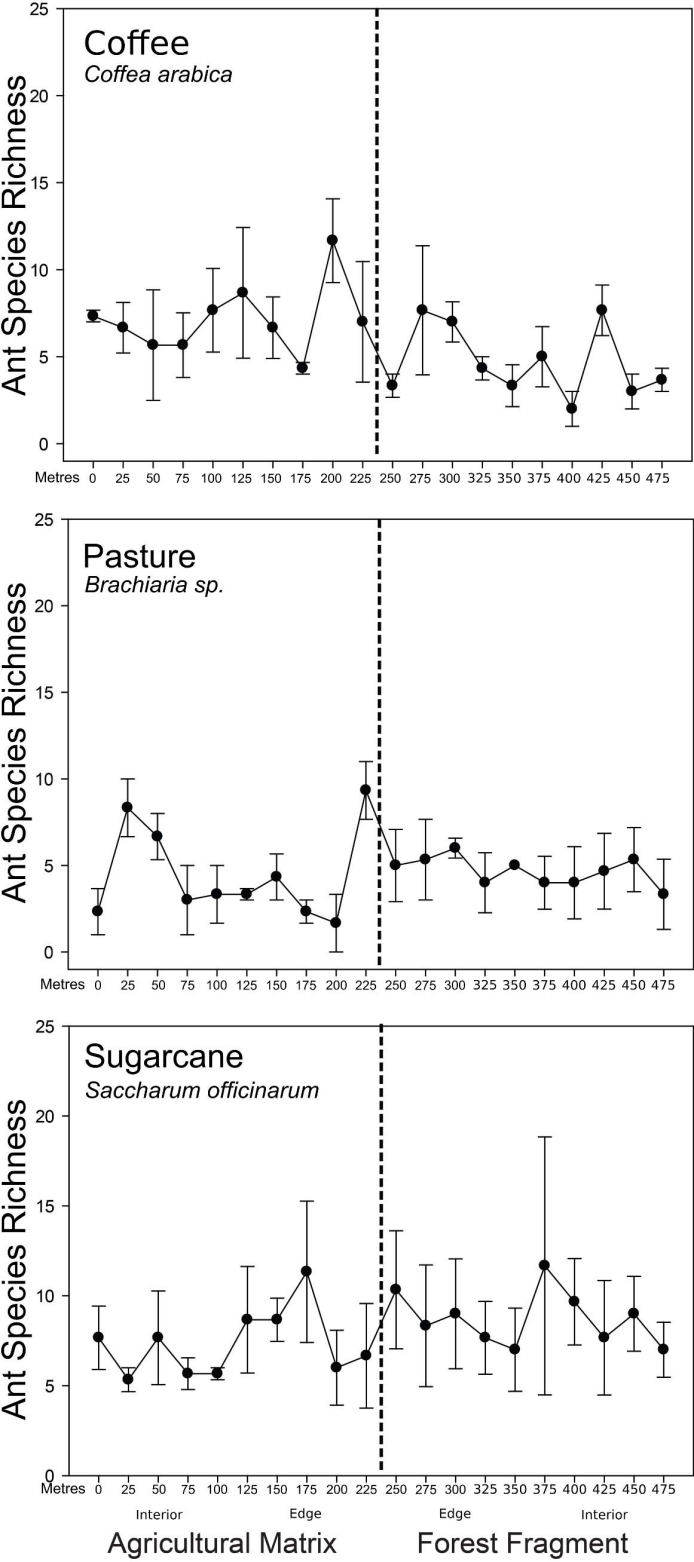
Variations in ant species richness in the systems.

**Fig 3:**
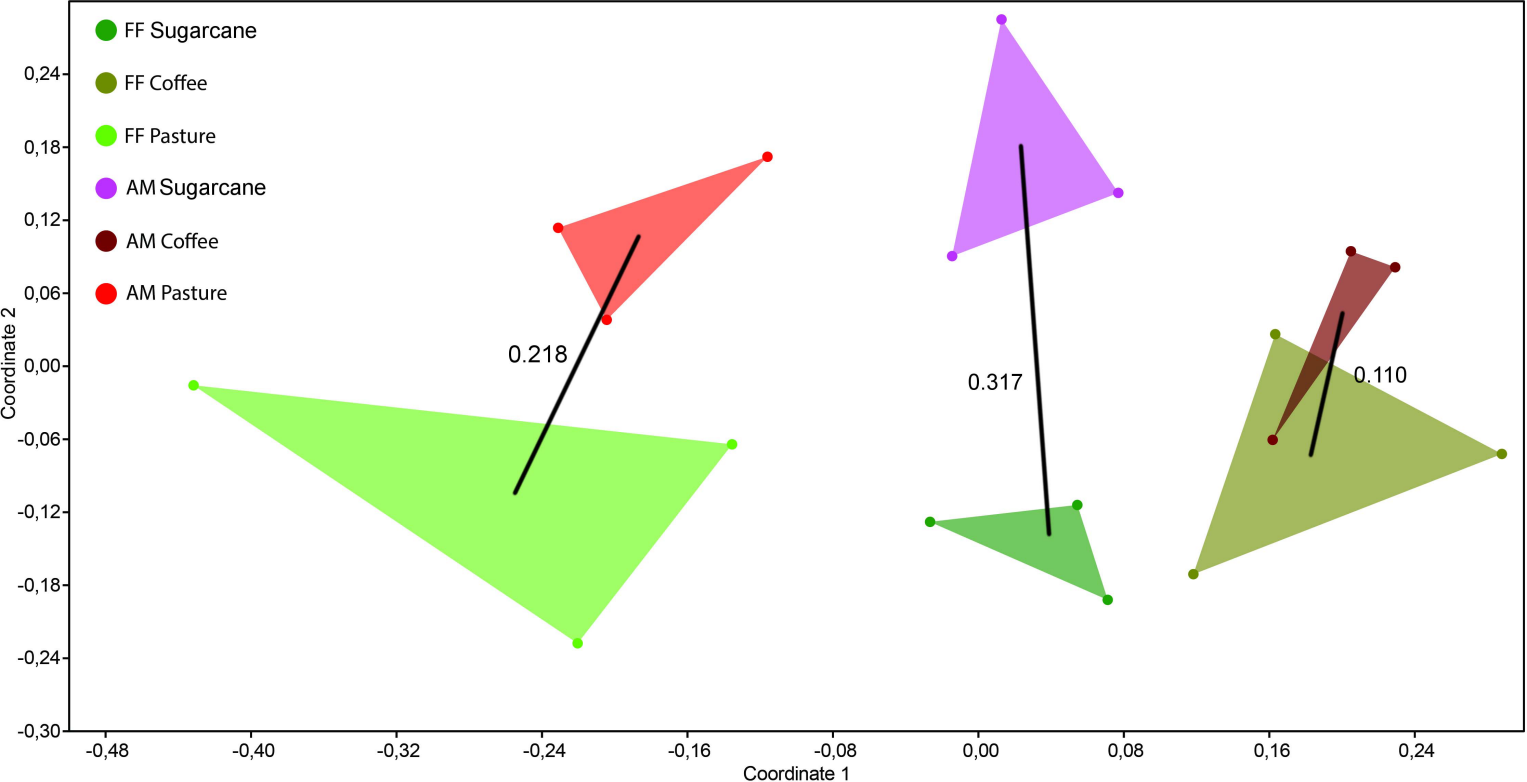
Non-metric dimensional scale plot. NMDS for the systems (FF—forest fragment and AM—agricultural matrix). The black lines and numbers represent Jaccard’s similarity values.

The sampled sites had an influence on ant species richness (Θ = 9.413; log-likelihood: -462.800; AIC: 941.600; χ^2^ = 13.137; *df* = 5; p = 0.022) (Fig 2). The post-hoc analysis shows that ant richness in the locations of pasture and sugarcane are different (Tukey: Z = -1.964, p = 0.049); in addition, the forest fragments in direct contact with pasture were different from forest fragments surrounded by sugarcane (Tukey: Z = -2.473, p = 0.013) and fragments surrounded by coffee (Tukey: Z = -2.412, p = 0.015). We also found that each location had a characteristic ant richness, with significant differences among ant assemblages (PERMANOVA: pseudo-*F* = 1.913, *df* = 5, p < 0.001; PERMDISP: F = 0.331, *df* = 5, p = 0.885 [i.e., our data has homogeneous variance]). The similarity analysis between locations showed that there was a low similarity between the ant communities (Table 3). The nMDS showed a separation of the ant community composition between the locations (Fig 3). The stress of analysis was 0.19, which indicates a good representation of our data in a multidimensional space [61]. The values of R^2^ = 0.936 and ‘fit-based’ R^2^ = 0.925 indicate the goodness of fit of our model [49]. In the ordination analysis, agricultural matrices of the same type remained closer; in a similar way, the forest fragments surrounded by the same matrix type remained closer than others surrounding by a different agricultural matrices. The similarity analysis among the agricultural matrices showed a reasonably high similarity between coffee matrix and forest fragments surrounded by coffee, although not significantly so (p = 0.308 [1-Diss_Jaccard_]) (Fig 3).

**Table 3:** Jaccard’s index of similarity among the systems. (FF–forest fragment, AM–agricultural matrix). Numbers are rounded to three decimal places. The values range from 0 (not similar) to 1(totally similar). Diagonal values are the number of unique species for the locations. Upper diagonal shows the number of species that occur in both sites.

Locations	FF Sugarcane	FF Pasture	FF Coffee	AM Sugarcane	AM Pasture	AM Coffee
FF Sugarcane	**25**	20	27	21	19	21
FF Pasture	0.189	**7**	15	18	22	16
FF Coffee	0.250	0.160	**11**	25	10	28
AM Sugarcane	0.187	0.202	0.275	**12**	19	23
AM Pasture	0.174	0.275	0.099	0.211	**12**	16
AM Coffee	0.183	0.170	0.308	0.245	0.167	**15**

**Fig 4:**
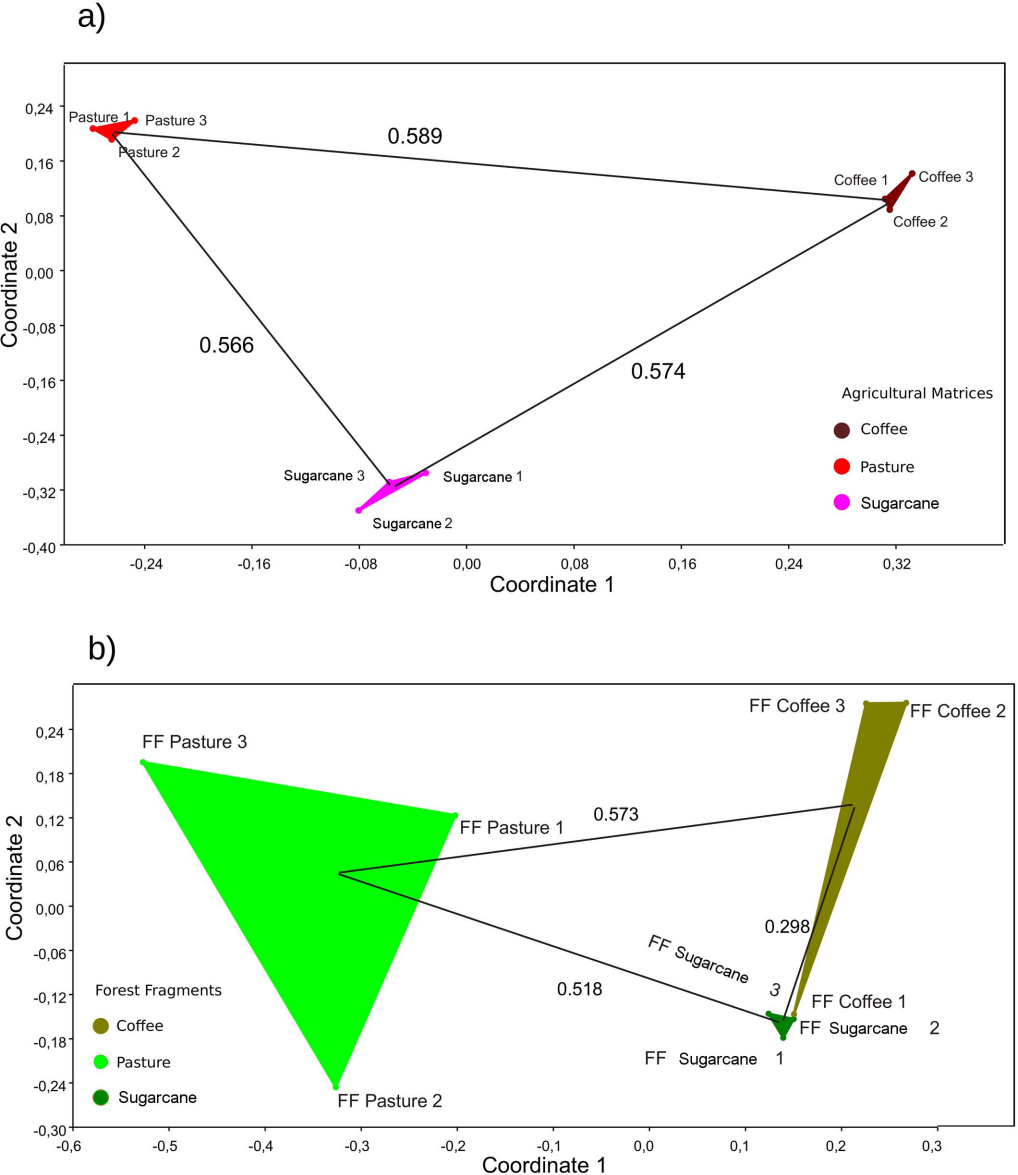
Non-metric dimensional scale plot. A) NMDS for agricultural matrix; B) NMDS for forest fragment. The black lines and numbers represent Jaccard’s similarity values.

We found a difference in ant species richness among the transect categories (AIC: 949.311; χ^2^ = 41.928; *df* = 11; p < 0.001). There was a difference between the interior of agricultural matrix of pasture and sugarcane (Tukey: Z = -3.145; p = 0.014), and pasture and coffee (Tukey: Z = -2.776; p = 0.027). In addition, there were differences between interior of forest fragments of pasture and sugarcane (Tukey: Z = -3.501; p = 0.010), and interior of forest fragment of coffee and interior of forest fragment of sugarcane (Tukey: Z = -3.501; p = 0.010). Likewise, we found a difference between the interior of the agricultural matrix of coffee and interior of the forest fragment embedded in coffee (Tukey: Z = -2.711; p = 0.027). Ant species richness among the points fluctuated and then, generally, stabilized in the forest fragment (Fig 4). Otherwise, we found differences in the diversity of the four transect categories (PERMANOVA: pseudo-*F* = 1.830, *df* = 11, p < 0.001; PERMDISP: F = 0.339, *df* = 11, p = 0.973 [i.e., our data has homogeneous variance]). The similarity analysis showed that the major similarity was among matrices of the same type. The pasture matrices were more similar among themselves than other matrices or forest fragments.

**Fig 5:**
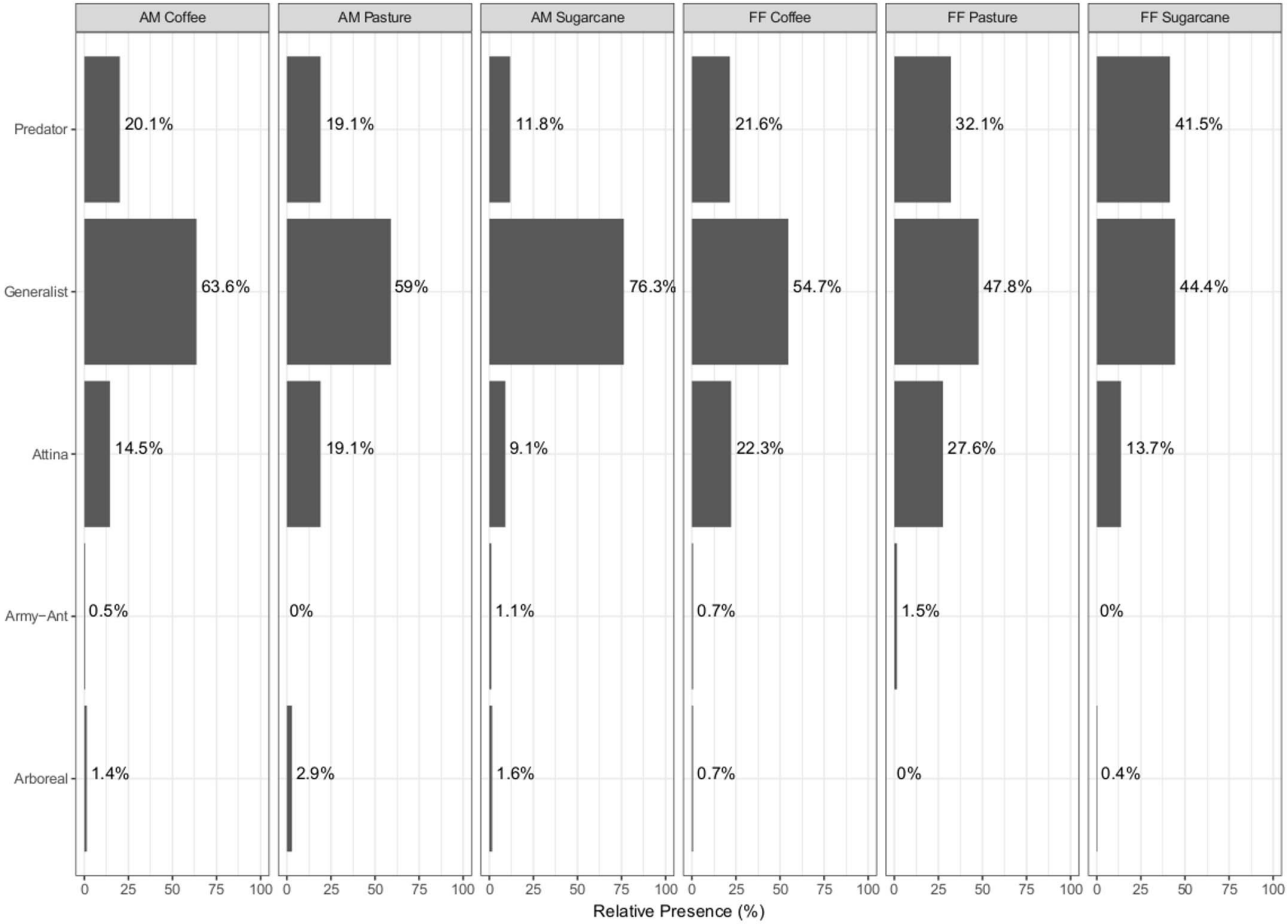
Functional guilds of ants present in the systems (FF—forest fragments and AM—agricultural matrices).

Generalist was the most commonly registered guild, and members of this guild were more abundant in the matrices (66.32%) than in the forest fragments (48.83%). More representatives from the predator guild were recorded in the forest fragments (32.03%) than in the matrices (16.67%). Arboreal ants were more abundant in the pastures (2.89%). However, we did not find arboreal ants in the forest fragments surrounded by pasture. Army ants were more abundant in forest fragments surrounded by pastures (1.5%) and were not found in pastures. We recorded more Attina guild species in the forest fragments than in the matrices (Fig 5). There were differences in the abundance of ants within functional guilds between most locations, with a few exceptions that did not represent any consistent trends (G = 102.35, df = 20, p < 0.001) (Table 4). The results of G Test analysis among the sites showed that the abundance of the predator and generalist ant guilds were different to the other guilds (G Test: predators: G = 83.041; p < 0.001; generalists: G = 47.916; p < 0.001).

**Table 4:** Pairwise G test comparing functional guilds between locations.

	FF Sugarcane	FF Pasture	FF Coffee	AM Sugarcane	AM Pasture	AM Coffee
FF Sugarcane		G = 22.851	G = 19.026	G = 61.751	G = 27.613	G = 28.223
FF Pasture	< 0.001*		G = 3.211	G = 35.077	G = 13.804	G = 14.938
FF Coffee	< 0.001*	0.523		G = 20.243	G = 4.601	G = 4.532
AM Sugarcane	< 0.001*	< 0.001*	< 0.001*		G = 16.820	G = 9.559
AM Pasture	< 0.001*	0.008*	0.331	0.002*		G = 3.796
AM Coffee	< 0.001*	0.005*	0.339	0.048*	0.434	

* means significant difference among the composition of functional guilds. The p values are in the lower diagonal and, the G values are in the upper diagonal.

## Discussion

According to our results, the agricultural matrices that we studied exert an influence on the embedded forest fragments and this affects ant community richness and composition. The results of the similarity analyses and the differences in the proportion of the functional guilds on each site support this statement.

Ant species richness varied among the locations. *Pheidole* was the most represented genus in our samples, being found in all locations. This representativeness could be due to it being a hyperdiverse, cosmopolitan genus of generalist ants which can use several types of resources [36,38]. On the other hand, the most abundant individuals belong to the genus *Atta* (26.13%). Ants of this genus build giant nests [62] with up to eight million workers [36]; we believe that higher abundance can be a reflection of number of workers in colonies (or colony) near to pitfall traps.

We found difference between ant richness and composition in the sugarcane crops and pasture. The lower richness in the pastures can be explained by the environmental factors that act direct on the pasture, such as sun exposition and winds. The habitat exerts a pressure on the ant community and that pressure can shape the richness and composition in those locations [63]. Furthermore, there were difference between forest fragments surrounded by pastures and those surrounded by sugarcane, and forest fragments surrounded by coffee and forest fragments surrounded by sugarcane. These results corroborate our hypothesis that the agricultural matrices may apply pressure on the ant community inside in the forest fragment.

There were no differences in ant richness between locations (matrices and forest fragments surrounded by their respective agricultural crop, example Forest fragments surrounded by sugarcane and sugarcane crops). However, the communities were different among the sampled sites. Some species are very flexible and can survive in the homogeneous conditions provided in this agricultural matrix [11]. Other researchers have verified that habitat heterogeneity is a determinant factor of richness [19,63] and composition [17,63]of ant species. But, unlike the findings of these researchers, our findings indicate that habitat shapes the ant communities but does not influence species richness, in some locations. This may be because there can be an increase in the number of certain generalist species in disturbed environments [11,64]. In pasture, habitat homogeneity and low vegetation height are probably the main factors causing the loss of ant richness, albeit not significantly so. Our results are opposite to those of another study [65], which found that pasture matrices are richer than coffee matrices. We found that the pasture and coffee matrices had the same species richness but were different in species composition.

These results for richness could be related to the turnover of ant species in the assemblages [20]. Species richness has limitations for measuring biodiversity, and is not necessarily sensitive to species turnover (both spatially and temporally) [20,21]. Similar results were found by Stork and colleagues [66] who found that species richness did not change from undisturbed to disturbed environments in seven of eight studied taxa. Our results present clear evidence that species richness is not a good indicator, when used alone, of local biodiversity and for not capturing changes in the species turnover. Using only richness in decision-making in priority areas of conservation could lead to misleading and less reliable decisions being made.

The similarity between the coffee matrices and the embedded forest fragments, albeit not extremely high, could be indicating that the coffee matrices can provide similar habitat features for ant species to those of forest fragments. The coffee matrices are characterized by having arboreal, and perennial vegetation, and this may confer permeability to the matrix, facilitating species movement [67]. This permeability could affect the community dynamics inside of forest fragments [5], influencing immigration and emigration among forest fragments [5,9,10] and therefore contributing to the maintenance of local biodiversity. In view of this, we propose that in these locations (coffee and the surrounding forest fragments) there is a low species movement (ie. between the forest fragment and agricultural matrix) in the community mediated by the vegetation composition.

On the other hand, the ant composition diverged between the edge and the interior of forest fragments surrounded by pasture matrices. As mentioned before, the composition and the vegetation type of the matrix can directly influence the forest fragment [2,68]. The agricultural matrix can provide protection against stochastic factors at the forest edge, reducing the edge effect [2,45,68]. This protection is generally facilitated by the portion of the agricultural matrix that is in contact with the forest edge and is dependent on the structure of the agricultural matrix [20,68,69]. We propose that this area be considered as a ‘pre-buffer zone’ in contrast to the ‘buffer-zone’ from the forest fragment edge [70]. The vegetation type of the ‘pre-buffer zone’ has been shown to be directly related to the high mortality of trees in Amazonia [68]; however, until now, there are no studies that show this also influences ant communities. In some cases, when the matrix management is intense (i.e. continuous pruning, application of herbicides and insecticides), this buffer zone is reduced or does not exist, which could cause abiotic factors to act directly on forest fragments, and therefore on ant communities in these areas [29].

Forest fragments surrounded by pasture are more influenced by the matrix compared to the other locations [20,69]. In several cases, the forest fragment surrounded by pasture is an extension of the original pasture, with cattle walking between the pasture into the forest fragment (Assis, D. S., personal observation). Based on this, we suggest that there are three main factors that shape ant communities in pastures and adjacent forest fragments, namely: (1) the use of a forest fragments as an extension of the pasture matrix; (2) the lack of a ‘pre-buffer zone’; and (3) invasive species arriving in pastures and spreading to forest fragments. In addition, we suggest that the ‘pre-buffer zone’ as an important factor in determining the ant assemblage, because when the vegetation in an agricultural matrix is continuously removed or altered by human activities, there is no ‘pre-buffer zone’ and all environmental stochastic factors act directly on the forest fragment.

Regarding the proportion of the functional guilds, the high number of generalist species in the agricultural matrices indicates that these species can survive and even dominate in homogeneous habitats [11,71], while heterogeneous habitats support a more balanced spread of functional guilds [71]. Agricultural matrices provide few resources compared to forest fragments, which puts pressure on the animal community [9,69]. This selective pressure acts on the species, and only those with a wide niche can survive in this type of environment [11,72]. Similar results have been found for birds [73] and amphibians [74].

In forest fragments, however, there were more predator species than agricultural matrices. We expected more predator species in heterogeneous environments [75]. Predatory ants experience more pressure than generalists because they are more sensitive to environmental changes [76] and, thus, are a better group to use when assessing the environmental impact on the habitat [76,77]. On the other hand, arboreal ants were more frequently recorded in pastures, probably because the lack of resources in these matrices forces the ants to forage on the ground. In general, the sampled pastures only had a few isolated trees. Therefore, it is likely that the environment where the ants live induces considerable competition and so they are forced to forage on the ground. Another hypothesis is that wind and/or rain throw the ants from the few surviving trees to the ground in the more exposed agricultural matrices.

We add adjacent vegetation type to Lawton's hypothesis [78] in which ant communities are shaped not only by vegetation structure where they are inserted but also by the surrounding vegetation, an issue that is supported by similar studies on ants in fragmented landscapes [17,19]. This would explain the divergence among the ant communities in different agricultural matrices and surrounding forest fragment, an important consideration for reconciling biodiversity conservation and agriculture throughout the landscape.

The findings of our work supplement those from similar studies elsewhere in the world [16–19] and provide important information for both conservation decisions and scientific knowledge in general. First, some agricultural matrices, such as coffee, can support a similar diversity of ants to the forest fragments. Then, adjacent agricultural matrices are the ‘gateway’ to invasive and opportunistic ants, and could increase the incidence of generalist species inside a forest fragment. In addition, the ant guild that best represents the changes in the environment is the predators. Land use, combined with inadequate management, are the major threats to biodiversity because they cause environmental homogenization and exert a constant pressure on the organisms therein. The type of management can cushion the impact of homogenization and mitigate the damage caused by it. Finally, we conclude that species composition is a better reflection than species richness of changes in the fragmented environment, although both measures should be used in a complementary approach to such assessments. To characterize these ant communities, we suggest using analysis of species composition and functional guilds together with richness and abundance.

## Supporting information

S1 Fig Rarefaction curve. Lines in red are agricultural matrices and the green lines are the forest fragments.
(DOCX)

S1 Table Numbers of each ant species sampled at the various locations. (XLSX)

## References

[1] Skole D, Tucker C. Tropical deforestation and habitat fragmentation in the Amazon: satellite data from 1978 to 1988. Science. 1993;260: 1905–1910. doi: 10.1126/science.260.5116.1905 17836720 10.1126/science.260.5116.1905

[2] Murcia C. Edge effects in fragmented forests: implications for conservation. Trends Ecol Evol. 1995;10: 58–62. doi: 10.1016/S0169-5347(00)88977-6 21236953 10.1016/S0169-5347(00)88977-6

[3] Fahrig L. Effects of habitat fragmentation on biodiversity. Annu Rev Ecol Evol Syst. 2003;34: 487–515. doi: 10.1146/annurev.ecolsys.34.011802.132419

[4] Fahrig L. Ecological responses to habitat fragmentation *per se*. Annu Rev Ecol Evol Syst. 2017;48: 1–23. doi: 10.1146/annurev-ecolsys-110316-022612

[5] Donald PF, Evans AD. Habitat connectivity and matrix restoration: the wider implications of agri-environment schemes. J Appl Ecol. 2006;43: 209–218. doi: 10.1111/j.1365-2664.2006.01146.x

[6] Estavillo C, Pardini R, da Rocha PLB. Forest loss and the biodiversity threshold: an evaluation considering species habitat requirements and the use of matrix habitats. PloS One. 2013;8: e82369 doi: 10.1371/journal.pone.0082369 24324776 10.1371/journal.pone.0082369 PMC3853156

[7] Kremen C, Williams NM, Bugg RL, Fay JP, Thorp RW. The area requirements of an ecosystem service: Crop pollination by native bee communities in California. Ecol Lett. 2004;7: 1109–1119. doi: 10.1111/j.1461-0248.2004.00662.x

[8] Chapin FS, Zavaleta ES, Eviner VT, Naylor RL, Vitousek PM, Reynolds HL, et al Consequences of changing biodiversity. Nature. 2000;405: 234–42. doi: 10.1038/35012241 10821284 10.1038/35012241

[9] Gascon C, Lovejoy T. Matrix habitat and species richness in tropical forest remnants. Biol Conserv. 1999;91: 223–229.

[10] Banks SC, Piggott MP, Stow AJ, Taylor AC. Sex and sociality in a disconnected world: a review of the impacts of habitat fragmentation on animal social interactions. Can J Zool. 2007;85: 1065–1079. doi: 10.1139/Z07-094

[11] McKinney M, Lockwood J. Biotic homogenization: a few winners replacing many losers in the next mass extinction. Trends Ecol Evol. 1999;14: 450–453. 10511724 10.1016/s0169-5347(99)01679-1

[12] Marasas M, Sarandón S., Cicchino A. Changes in soil arthropod functional group in a wheat crop under conventional and no tillage systems in Argentina. Appl Soil Ecol. 2001;18: 61–68. doi: 10.1016/S0929-1393(01)00148-2

[13] Deikumah JP, McAlpine C a, Maron M. Matrix intensification alters avian functional group composition in adjacent rainforest fragments. PLoS ONE. 2013;8: e74852 doi: 10.1371/journal.pone.0074852 24058634 10.1371/journal.pone.0074852 PMC3772896

[14] Altieri MA. The ecological role of biodiversity in agroecosystems. Agric Ecosyst Environ. 1999;74: 19–31. doi: 10.1016/S0167-8809(99)00028-6

[15] Olff H, Ritchie ME. Fragmented nature: consequences for biodiversity. Landsc Urban Plan. 2002;58: 83–92. doi: 10.1016/S0169-2046(01)00211-0

[16] Spiesman BJ, Cumming GS. Communities in context: the influences of multiscale environmental variation on local ant community structure. Landsc Ecol. 2008;23: 313–325. doi: 10.1007/s10980-007-9186-3

[17] García-Martínez MÁ, Valenzuela-González JE, Escobar-Sarria F, López-Barrera F, Castaño-Meneses G. The surrounding landscape influences the diversity of leaf-litter ants in riparian cloud forest remnants. PLOS ONE. 2017;12: e0172464 doi: 10.1371/journal.pone.0172464 28234948 10.1371/journal.pone.0172464 PMC5325296

[18] Tagwireyi P, Sullivan SMP. Riverine landscape patch heterogeneity drives riparian ant assemblages in the Scioto River Basin, USA. PLOS ONE. 2015;10: e0124807 doi: 10.1371/journal.pone.0124807 25894540 10.1371/journal.pone.0124807 PMC4403917

[19] De La Mora A, Murnen C, Philpott S. Local and landscape drivers of biodiversity of four groups of ants in coffee landscapes. Biodivers Conserv. 2013;22: 871–888. doi: 10.1007/s10531-013-0454-z

[20] Prevedello JA, Vieira MV. Does the type of matrix matter? A quantitative review of the evidence. Biodivers Conserv. 2010;19: 1205–1223. doi: 10.1007/s10531-009-9750-z

[21] Hillebrand H, Blasius B, Borer ET, Chase JM, Downing JA, Eriksson BK, et al Biodiversity change is uncoupled from species richness trends: Consequences for conservation and monitoring. J Appl Ecol. 2017;55: 169–184. doi: 10.1111/1365-2664.12959

[22] Melo MM, Silva CM, Barbosa CS, Morais MC, D’Anunciação PER, da Silva VX, et al Fragment edge and isolation affect the food web: effects on the strength of interactions among trophic guilds. Biota Neotropica. 2016;16 doi: 10.1590/1676-0611-BN-2015-0088

[23] Dormann CF, Schweiger O, Augenstein I, Bailey D, Billeter R, De Blust G, et al Effects of landscape structure and land-use intensity on similarity of plant and animal communities. Glob Ecol Biogeogr. 2007;16: 774–787. doi: 10.1111/j.1466-8238.2007.00344.x

[24] Lustig A, Stouffer DB, Doscher C, Worner SP. Landscape metrics as a framework to measure the effect of landscape structure on the spread of invasive insect species. Landsc Ecol. 2017;32: 2311–2325. doi: 10.1007/s10980-017-0570-3

[25] Kupfer J, Malanson G, Franklin S. Not seeing the ocean for the islands: the mediating influence of matrix-based processes on forest fragmentation effects. Glob Ecol Biogeogr. 2006;15: 8–20. doi: 10.1111/j.1466-822x.2006.00204.x

[26] Chen J, Franklin JF, Spies TA. Contrasting microclimates among clearcut, edge, and interior of old-growth Douglas-fir forest. Agric For Meteorol. 1993;63: 219–237. doi: 10.1016/0168-1923(93)90061-L

[27] Fischer J, Lindenmayer DB. Landscape modification and habitat fragmentation: a synthesis. Glob Ecol Biogeogr. 2007;16: 265–280. doi: 10.1111/j.1466-8238.2007.00287.x

[28] Malcolm JAYR. Edge effects in central Amazonian Forest fragments. Ecology. 1994;75: 2438–2445.

[29] Gascon C, Williamson GB, Fonseca GAB da. Receding forest edges and vanishing reserves. Science. 2000;288: 1356–1358. doi: 10.1126/science.288.5470.1356 10847849 10.1126/science.288.5470.1356

[30] Didham RK, Lawton JH. Edge structure determines the magnitude of changes in microclimate and vegetation structure in tropical forest fragments. Biotropica. 1999;31: 17–30. doi: 10.2307/2663956

[31] Brown KS. Diversity, disturbance, and sustainable use of Neotropical forests: insects as indicators for conservation monitoring. J Insect Conserv. 1997;1: 25–42. doi: 10.1023/A:1018422807610

[32] Duelli P, Obrist MK. Biodiversity indicators: the choice of values and measures. Agric Ecosyst Environ. 2003;98: 87–98. doi: 10.1016/S0167-8809(03)00072-0

[33] Majer JD. Ants: Bio-indicators of minesite rehabilitation, land-use, and land conservation. Environ Manage. 1983;7: 375–383. doi: 10.1007/BF01866920

[34] Rosenberg DM, Danks HV, Lehmkuhl DM. Importance of insects in environmental impact assessment. Environ Manage. 1986;10: 773–783. doi: 10.1007/BF01867730

[35] Folgarait P. Ant biodiversity and its relationship to ecosystem functioning: A review. Biodivers Conserv. 1998;7: 1221–1244.

[36] Hölldobler BW, Wilson E. The Ants. Belknap Press of Harvard University Press; 1990.

[37] Fittkau EJE, Klinge H. On biomass and trophic structure of the central Amazonian Rain Forest ecosystem. Biotropica. 1973;5: 2–14.

[38] Delabie J, Agosti D, Nascimento I. Litter ant communities of the Brazilian Atlantic rain forest region In: Agosti D, Majer JD, Alonso L, Schultz TR, editors. Ground-dwelling Ants: case studies from the worlds rain forests. Australia: Curtin University of Technology School of Environmental Biology; 2000 pp. 1–18.

[39] Andersen AN. Functional groups and pattens of organization in north American ant communities: a comparison with Australia. J Biogeogr. 1997;24: 433–460. doi: 10.1111/j.1365-2699.1997.00137.x

[40] SOS Mata Atlântica. Atlas dos remanescentes florestais da Mata Atlântica—Período 2012–2013. 2014. Available: http://www.sosma.org.br/wp-content/uploads/2014/05/atlas_2012 2013_relatorio_tecnico_20141.pdf

[41] Gomes APC, Carvalho MM, Evangelista AR, Curi N, Araújo JGF, Pozza AAA, et al Biodiversidade em Minas Gerais: um atlas para sua conservacao. Summa Phytopathol Bras. 2005, 261, 29–34.

[42] Olivetti D, Mincato RL, Ayer JEB, Silva MLN, Curi N. Spatial and temporal modeling of water erosion in dystrophic red latosol (oxisol) used for farming and cattle raising activities in a sub-basin in the south of Minas Gerais. Ciênc E Agrotecnologia. 2015;39: 58–67. doi: 10.1590/S1413-70542015000100007

[43] Carneiro MS, Campos CCF, Beijo LA, Ramos FN. Anthropogenic matrices favor homogenization of tree reproductive functions in a highly fragmented landscape. PLOS ONE. 2016;11: e0164814 doi: 10.1371/journal.pone.0164814 27760218 10.1371/journal.pone.0164814 PMC5070737

[44] Sabu TK, Shiju RT. Efficacy of pitfall trapping, Winkler and Berlese extraction methods for measuring ground-dwelling arthropods in moist-deciduous forests in the Western Ghats. J Insect Sci Online. 2010;10: 98 doi: 10.1673/031.010.9801 20673122 10.1673/031.010.9801 PMC3383433

[45] Chen J, Franklin JF, Spies TA. Growing-season microclimatic gradients from clearcut edges into old-growth Douglas-Fir forests. Ecol Appl. 1995;5: 74–86. doi: 10.2307/1942053

[46] Bolton B. Identification guide to the ant genera of the world. Harvard University Press, 1994

[47] Bolton B. New general catalogue of the ants of the world. Harvard University Press; 1995.

[48] AntWeb [Internet]. Available: http://www.antweb.org/

[49] Oksanen J, Blanchet FG, Kindt R, Legendre P, Minchin PR, O’hara R, et al. The vegan package. 2017.

[50] R Foundation for Statistical Computing. R: A language and environment for statistical computing. Viena, Austria: R Foundation for Statistical Computing; 2016.

[51] Bates D, Sarkar D, Bates MD, Matrix L. The lme4 Package. October. 2007;2: 1–6. doi: 10.18637/jss.v067.i01

[52] Bolker B. R Development Core Team. bbmle: Tools for general maximum likelihood estimation. R package version 1.0. 18. 2016. 2015.

[53] Bretz F, Hothorn T, Westfall P. Multiple comparisons using R. CRC Press; 2016.

[54] Borcard D, Gillet F, Legendre P. Numerical ecology with R. New York, NY: Springer New York; 2011 doi: 10.1007/978-1-4419-7976-6

[55] Legendre P, Legendre L. Numerical ecology. Elsevier; 2012.

[56] Anderson MJ. A new method for non-parametric multivariate analysis of variance. Austral Ecol. 2001;26: 32–46. doi: 10.1111/j.1442-9993.2001.01070.pp.x

[57] Anderson MJ. Distance-based tests for homogeneity of multivariate dispersions. Biometrics. 2006;62: 245–253. doi: 10.1111/j.1541-0420.2005.00440.x 16542252 10.1111/j.1541-0420.2005.00440.x

[58] M Anderson. Permutational multivariate analysis of variance (PERMANOVA). Wiley StatsRef Stat Ref Online. 2017; doi: 10.1002/9781118445112.stat07841

[59] Gotelli NJ, Ellison AM. A primer of ecological statistics. ArtMed; 2004.

[60] Hervé M. RVAideMemoire: Diverse basic statistical and graphical functions. 2017. Available: https://CRAN.R-project.org/package = RVAideMemoire

[61] Clarke KR. Non-parametric multivariate analyses of changes in community structure. Aust J Ecol. 1993;18: 117–143. doi: 10.1111/j.1442-9993.1993.tb00438.x

[62] Moreira A, Forti L, Boaretto M, Andrade A, Lopes J, Ramos V. External and internal structure of *Atta bisphaerica* Forel (Hymenoptera: Formicidae) nests. J Appl Entomol. 2004;128: 204–211. doi: 10.1111/j.1439-0418.2004.00839.x

[63] Lassau S, Hochuli D. Effects of habitat complexity on ant assemblages. Ecography. 2004;27: 157–164.

[64] Dukes JS, Mooney HA. Does global change increase the success of biological invaders? Trends Ecol Evol. 1999;14: 135–139. doi: 10.1016/S0169-5347(98)01554-7 10322518 10.1016/s0169-5347(98)01554-7

[65] Dias N, Zanetti R, Santos M. Interação de fragmentos florestais com agroecossistemas adjacentes de café e pastagem: respostas das comunidades de formigas (Hymenoptera, Formicidae). Iheringia Sér Zool. 2008;98: 136–142.

[66] Stork NE., Srivastava DS, Eggleton P, Hodda M, Lawson G, Leakey RRB, et al Consistency of effects of tropical-forest disturbance on species composition and richness relative to use of indicator taxa. Conserv Biol. 2017;31: 924–933. doi: 10.1111/cobi.12883 27982481 10.1111/cobi.12883

[67] Muriel SB, Kattan GH. Effects of patch size and type of coffee matrix on Ithomiine butterfly diversity and dispersal in Cloud-Forest fragments. Conserv Biol. 2009;23: 948–956. doi: 10.1111/j.1523-1739.2009.01213.x 19627322 10.1111/j.1523-1739.2009.01213.x

[68] Mesquita RCG, Delamônica P, Laurance WF. Effect of surrounding vegetation on edge-related tree mortality in Amazonian forest fragments. Biol Conserv. 1999;91: 129–134. doi: 10.1016/S0006-3207(99)00086-5

[69] Ricketts TH. The matrix matters: effective isolation in fragmented landscapes. Am Nat. 2001;158: 87–99. doi: 10.1086/320863 18707317 10.1086/320863

[70] Magura T, Lövei GL, Tóthmérész B. Edge responses are different in edges under natural versus anthropogenic influence: a meta‐analysis using ground beetles. Ecol Evol. 2017;7: 1009–1017. doi: 10.1002/ece3.2722 28168036 10.1002/ece3.2722 PMC5288263

[71] Olden JD, Poff NL, Douglas MR, Douglas ME, Fausch KD. Ecological and evolutionary consequences of biotic homogenization. Trends Ecol Evol. 2004;19: 18–24. doi: 10.1016/j.tree.2003.09.010 16701221 10.1016/j.tree.2003.09.010

[72] Kassen R. The experimental evolution of specialists, generalists, and the maintenance of diversity. J Evol Biol. 2002;15: 173–190. doi: 10.1046/j.1420-9101.2002.00377.x

[73] Devictor V, Julliard R, Couvet D, Lee A, Jiguet F. Functional homogenization effect of urbanization on bird communities. Conserv Biol J Soc Conserv Biol. 2007;21: 741–51. doi: 10.1111/j.1523-1739.2007.00671.x 17531052 10.1111/j.1523-1739.2007.00671.x

[74] D’Anunciação PER, Silva MFV, Ferrante L, Assis DS, Casagrande T, Coelho AZG, et al Forest fragments surrounded by sugar cane are more inhospitable to terrestrial amphibian abundance than fragments surrounded by pasture. Int J Ecol. 2013;2013: 1–8. doi: 10.1155/2013/183726

[75] Philpott SM, Perfecto I, Vandermeer J. Effects of predatory ants on lower trophic levels across a gradient of coffee management complexity. J Anim Ecol. 2008;77: 505–11. doi: 10.1111/j.1365-2656.2008.01358.x 18248385 10.1111/j.1365-2656.2008.01358.x

[76] Dias NDS, Zanetti R, Santos MS, Peñaflor MFGV, Broglio SMF, Delabie JHC. The impact of coffee and pasture agriculture on predatory and omnivorous leaf-litter ants. J Insect Sci Online. 2013;13: 29 doi: 10.1673/031.013.2901 23902334 10.1673/031.013.2901 PMC3735050

[77] Wiescher PT, Pearce-Duvet JMC, Feener DH. Assembling an ant community: species functional traits reflect environmental filtering. Oecologia. 2012;169: 1063–1074. doi: 10.1007/s00442-012-2262-7 22294027 10.1007/s00442-012-2262-7

[78] Lawton JH. Plant architecture and the diversity of phytophagous insects. Annu Rev Entomol. 1983;28: 23–39. doi: 10.1146/annurev.en.28.010183.000323

