# Supplementary material for: Agricultural matrices affect ground ant assemblage composition inside forest fragments
Source: PLoS One. 2018 May 23;13(5):e0197697. doi: 10.1371/journal.pone.0197697 (PMC5965890; doi:10.1371/journal.pone.0197697)
Supplement: S1 Fig — Lines in red are agricultural matrices and the green lines are the forest fragments. (DOCX) [file pone.0197697.s001.docx]

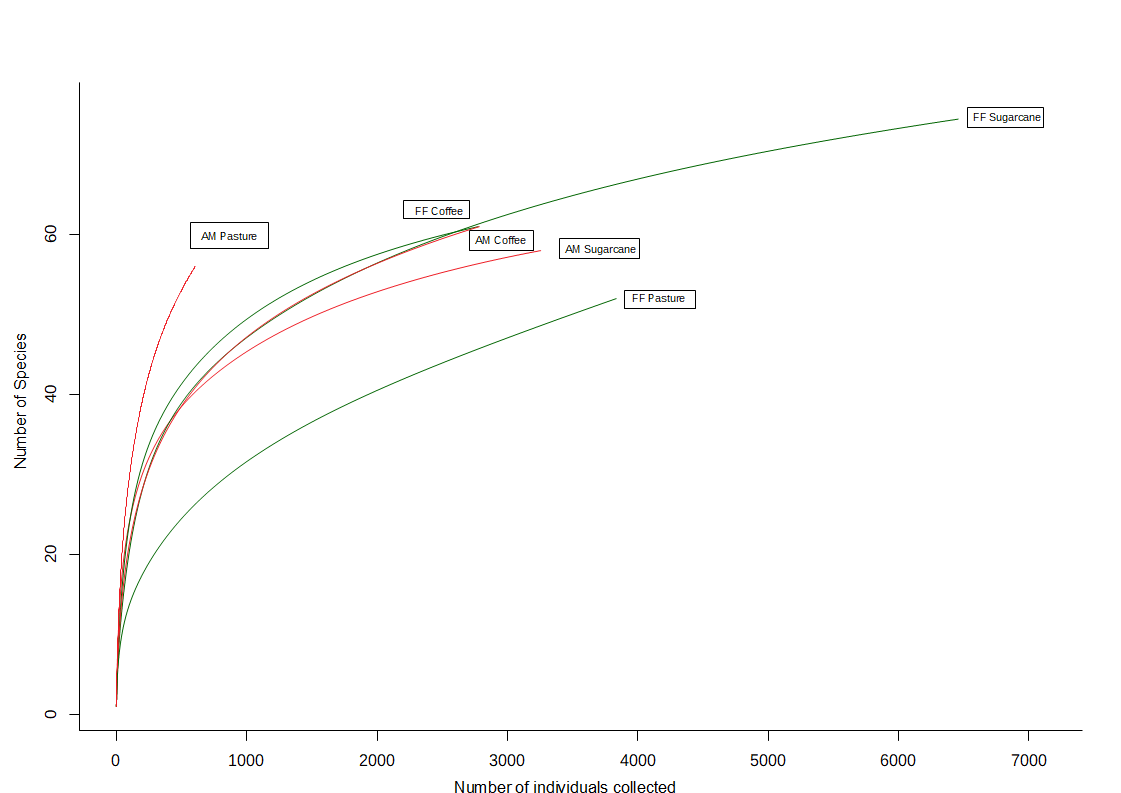


S1 Fig. Rarefaction curve. Lines in red are agricultural matrices and the green lines are the forest fragments.
